# Quality of life in diverticulosis across different clinical manifestations: Findings from the DIVIPACT cohort including 19,244 patients

**DOI:** 10.1111/codi.70681

**Published:** 2026-11-12

**Authors:** Helene R. Dalby, Rune Erichsen, Katrine J. Emmertsen

**Affiliations:** ^1^ Department of Clinical Medicine University Clinic for Coloproctology and Endoscopy (U‐COPE), Aarhus University Aarhus Denmark; ^2^ Department of Surgery Viborg Regional Hospital Viborg Denmark; ^3^ Department of Surgery Randers Regional Hospital Randers Denmark

**Keywords:** diverticular disease, diverticulitis, diverticulosis, quality of life, registry data, survey

## Abstract

**Aim:**

This study aimed to examine disease‐specific quality of life (QoL) among individuals with diverticulosis across different clinical manifestations and identify characteristics associated with an unacceptable symptom burden.

**Method:**

This was a population‐based study within the DIVIPACT cohort, linking cross‐sectional questionnaire data collected in 2023 with Danish health registry data collected from 2010 to 2024. The DIVIPACT cohort comprises 19,244 subjects diagnosed with diverticulosis (K572‐9) in the Central Denmark Region (~1.3 million residents) from 2010 to 2022. Respondents were classified into mutually exclusive groups according to prior hospital management for diverticulosis (emergency resection, elective resection, laparoscopic lavage, inpatient, outpatient or diverticulosis only as a secondary diagnosis) and characterised based on registry and self‐reported data. QoL was evaluated using the Diverticular Disease Quality of Life (DV‐QoL) instrument.

**Results:**

The total DV‐QoL was completed by 17,056 respondents (89% of all). An unacceptable symptom burden (i.e. total DV‐QoL score ≥3.2) was reported by 53% of patients in the emergency resection group, 62% in the elective resection group, 42% in the laparoscopic lavage group, 42% in the inpatient group, 32% in the outpatient group, and 24% in the diverticulosis group. Female sex, younger age and reduced self‐rated performance status were associated with an unacceptable symptom burden. Compared with the diverticulosis group, all hospital‐managed groups demonstrated higher odds of an unacceptable symptom burden.

**Conclusion:**

Diverticular disease is associated with a substantial symptom burden that extends beyond acute clinical episodes and is not fully captured by traditional disease classifications. Demographic and functional factors play a central role in perceived disease burden.

**What does this paper add to the literature?**

Quality of life across different clinical manifestations of diverticular disease remains poorly characterised since previous studies have focused on hospitalised or surgically treated patients. This study shows that substantial disease‐specific symptom burden extends beyond acute clinical episodes and identifies associated patient characteristics. These findings support more holistic, patient‐centred management.

## INTRODUCTION

Current guidelines on the management of diverticular disease emphasise quality of life (QoL) as an essential consideration alongside traditional clinical outcomes [1, 2]. In chronic gastrointestinal conditions, symptoms and their consequences for physical, psychological and social functioning may substantially affect patients' health‐related QoL, making patient‐reported outcomes an important complement to conventional clinical endpoints [3]. However, most existing studies assessing QoL in diverticulosis have focused on patients admitted with acute diverticulitis or those undergoing surgery for chronic diverticulitis [4, 5, 6, 7, 8, 9, 10, 11, 12, 13, 14, 15]. Consequently, the available evidence largely reflects patients with more clinically apparent disease.

Moreover, the psychosocial and functional consequences of diverticulosis remain poorly characterised, particularly among individuals without previous hospital admission for acute diverticulitis. Although diverticulosis is often considered an incidental and largely asymptomatic finding, emerging evidence suggests that its impact may extend beyond acute episodes of diverticulitis. The DIVIPACT cohort demonstrated that bowel function affected QoL in 44% of individuals with diverticulosis without previous hospital treatment [16]. Furthermore, recent validation of the disease‐specific diverticulitis quality‐of‐life instrument (DV‐QOL) in a large population‐based cohort showed that 24% of individuals with diverticulosis had an unacceptable symptom state [17]. Consequently, knowledge regarding the overall long‐term psychosocial burden of diverticulosis across the disease spectrum is lacking. It also remains unclear which patient characteristics are associated with an unacceptable symptom state. Addressing these gaps is essential to inform more comprehensive, patient‐centred management strategies.

The DIVIPACT cohort constitutes a large, population‐based cohort designed to investigate the impact of diverticulosis on daily life [16]. The present study aimed to evaluate both generic and disease‐specific QoL among individuals with diverticulosis across different clinical manifestations and identify patient characteristics associated with an unacceptable symptom burden.

## MATERIALS AND METHODS

### Study design

The DIVIPACT cohort comprises patients diagnosed with diverticulosis in the Central Denmark Region from 2010 to 2022 who responded to a cross‐sectional online questionnaire survey conducted in April–May 2023 [16]. Written informed consent was obtained from all respondents. Patients were asked for permission to link questionnaire data to medical record data. The study was registered at the Danish Data Protection Agency in the Central Denmark Region (record no. 1‐16‐02‐61‐22), and the disclosure of patient information was approved by the Central Denmark Region (record no. 1‐45‐70‐19‐22). This study is reported in accordance with The Strengthening in Reporting of Observational Studies in Epidemiology (STROBE) Statement guidelines for reporting cross‐sectional studies [18].

Registry data collected from 2010 to 2024 were obtained from the Business Intelligence (BI) Data Warehouse, which stores regionally collected medical record data in the Central Denmark Region (5 public hospitals, ~1.3 million residents), aligning with national registries known to have high validity and completeness [19, 20]. The unique personal civil registration (CPR) number assigned to all residents in Denmark enables unambiguous individual‐level data linkage.

### Study cohort

Invited subjects included residents with a diagnosis of diverticulosis (ICD10 codes K57.2‐9) recorded as either a primary or secondary diagnosis at an inpatient or outpatient hospital contact in the Central Denmark Region between 2010 and 2022. Exclusion criteria were colorectal cancer, dementia, death before survey distribution, and exemption from receiving digital mail from the public Danish authorities.

Based on registry data, respondents were classified in mutually exclusive groups according to the following hierarchy of prior hospital management for diverticulosis:

1. *emergency colonic resection* for diverticular disease,
2. *elective colonic resection* for diverticular disease,
3. *laparoscopic peritoneal lavage* for diverticular disease,
4. *inpatient* contact with diverticulosis as the primary diagnosis,
5. *outpatient* contact with diverticulosis as the primary diagnosis, and
6. *diverticulosis* recorded solely as a secondary diagnosis in any hospital contact.

Information on comorbidity was obtained from registry data and classified according to the Charlson Comorbidity Index (CCI) based on all diagnoses recorded before survey completion [21]. In addition, the number of hospital contacts with diverticulosis as the primary diagnosis and the time from the first registered diverticulosis diagnosis to survey completion were extracted.

### Quality of life measurements

In addition to several ad hoc questions on health factors and daily life, QoL was assessed using two validated instruments that measured generic and disease‐specific QoL, respectively, both of which were validated in Danish‐speaking cohorts.

Generic QoL was assessed by the EuroQol visual analogue scale (EQ‐VAS) [22] which assesses the individual patient's evaluation of their own current health state on a scale from 0 (worst) to 100 (best).

Disease‐specific QoL was assessed by the diverticulitis QoL (DV‐QoL) instrument [3], which has been validated as a reliable tool for measuring QoL in the DIVIPACT cohort [17]. The instrument contains 17 items covering four domains: *physical symptoms* (5 items), *concerns* (3 items), *feelings* (4 items), and *behavioural changes* (5 items) and is available in the Supporting Information. The score for each domain is calculated by normalising the sum of the relevant items on a 0‐to‐10 scale, with lower scores indicating a better QoL. The DV‐QoL total score is calculated by summing the normalised scores of the four domains on a 0‐to‐10 scale, with each domain weighted equally. The patient's acceptable symptom state (PASS) score has been established at 3.2, that is, scores ≥3.2 indicate an unacceptable symptom burden, hence QoL‐impacting disease [23].

### Statistical analysis

Descriptive statistics were compiled to characterise the respondents using both medical record data and self‐reported health variables. Quantitative data were summarised as medians with interquartile ranges (IQRs), and categorical data as absolute numbers and percentages. Groups were compared using Pearson's chi‐squared test for categorical variables and the Kruskal–Wallis rank‐sum test for continuous variables, as appropriate.

To identify factors associated with an unacceptable symptom burden, univariate and multivariate logistic regression models were fitted. PASS was analysed as a dichotomous variable. The multivariable model included age group, sex and hospital management group, selected a priori based on their role as key demographic and clinical characteristics. Variables that may lie on the causal pathway between diverticular disease and symptom burden, including performance status and comorbidity measures, were not included in the adjusted model to minimise the risk of overadjustment. Odds ratios (ORs) with 95% confidence intervals (CIs) were derived from exponentiated coefficients.

### Missing data

Proportions for questionnaire items were calculated using item‐specific response rates to account for partial responses. DV‐QoL scores were only calculated for respondents who had completed all items of the instrument.

Statistical analyses were conducted using RStudio, version 2024.04.2 + 764 (Posit PBC).

## RESULTS

### Cohort characteristics

Of 28,329 subjects invited to participate in the survey, 20,961 (74%) responded, and 19,244 (92% of respondents) consented to data linkage. Of those consenting to data linkage, 166 (1%) had undergone emergency colonic resection, 304 (2%) had undergone elective colonic resection, 190 (1%) had undergone laparoscopic peritoneal lavage without subsequent colonic resection, 3528 (18%) were classified as inpatients, 8666 (45%) as outpatients, and 6394 (33%) as having diverticulosis. The median time from diagnosis to survey completion was 4 years (IQR: 2–7), ranging from 3 years in the diverticulosis group to 7 years in the elective resection group (Table 1). The median age was lowest in the laparoscopic lavage group (62 years) and highest in the diverticulosis group (72 years). The proportion of women was highest among patients who had undergone resection and among inpatients. Medical records data and self‐reported health variables are presented in Table 1. Among patients who underwent colonic resection, 49 had previously undergone laparoscopic lavage; 23 (46.9%) subsequently underwent emergency resection and 26 (53.1%) elective resection.

**TABLE 1:** Characteristics of subjects in the DIVIPACT cohort.

	Overall 19,244	Emergency resection 166 (1%)	Elective resection 304 (2%)	Lap. Lavage 190 (1%)	Inpatient 3528 (18%)	Outpatient 8663 (45%)	Diverticulosis 6393 (33%)
Age	70 (63–77)	69 (59–75)	66 (59–73)	62 (53–70)	66 (58–74)	71 (64–77)	72 (65–77)
Sex							
Female	9922 (52)	96 (58)	189 (62)	95 (50)	2221 (63)	4477 (52)	2844 (44)
Male	9322 (48)	70 (42)	115 (38)	95 (50)	1307 (37)	4186 (48)	3549 (56)
CCI score							
0	10,952 (57)	86 (52)	176 (58)	120 (63)	2157 (61)	4877 (56)	3536 (55)
1–2	5761 (30)	58 (35)	97 (32)	53 (28)	960 (27)	2644 (31)	1949 (30)
3+	2531 (13)	22 (13)	31 (10)	17 (8.9)	411 (12)	1142 (13)	908 (14)
Hospital contacts due to diverticulosis before survey completion							
0	6389 (33)	0	0	0	0	0	6389 (100)
1	8111 (42)	29 (17)	6 (2.0)	49 (26)	898 (25)	7125 (82)	0
2–3	3547 (18)	35 (21)	34 (11)	87 (46)	1993 (56)	1398 (16)	0
4+	1168 (6.1)	102 (61)	264 (87)	54 (28)	636 (18)	112 (1.3)	0
Time, diagnosis to survey (years)	4.0 (2–7)	6 (2–9)	7 (4–9)	6 (3–8)	5 (3–8)	4 (2–7)	3 (2–6)
Self‐reported data from survey							
*Stoma* *							
Current	78 (0.4)	49 (30)	29 (10)	–	–	–	–
Reversed	78 (0.4)	56 (34)	22 (7)	–	–	–	–
Never	19,088 (99)	61 (37)	253 (83)	190 (100)	3528 (99)	8663 (100)	6393 (100)
BMI (kg/m^2^) (*n* = 16,868)	26.8 (24.2–30.1)	26.7 (24.1–29.6)	27.2 (24.8–30.1)	28.0 (24.9–31.4)	27.6 (24.6–31.1)	26.5 (23.9–29.7)	26.9 (24.3–30.1)
Smoking status (*n* = 17,260)							
Never	6897 (40)	50 (33)	93 (34)	77 (44)	1317 (41)	3191 (41)	2169 (38)
Former	8481 (49)	74 (49)	149 (55)	78 (44)	1540 (48)	3783 (49)	2857 (50)
Smoker	1882 (11)	26 (17)	30 (11)	21 (12)	354 (11)	789 (10)	662 (12)
Alcohol (units/week) (*n* = 17,167)							
<10	14,275 (83)	124 (84)	239 (88)	153 (87)	2794 (87)	6434 (83)	4531 (80)
≥10	2892 (17)	24 (16)	32 (12)	22 (13)	404 (13)	1291 (17)	1119 (20)
Physical activity (time/week) (*n* = 16,611)							
<15 min	2742 (17)	38 (27)	37 (16)	36 (21)	555 (18)	1200 (16)	876 (16)
15–120 min	7506 (45)	63 (45)	107 (47)	63 (38)	1410 (45)	3387 (45)	2476 (45)
≥120 min	6363 (38)	39 (28)	84 (37)	69 (41)	1137 (37)	2885 (39)	2149 (39)
Self‐reported performance status (*n* = 16,964)							
Fully active	9291 (55)	62 (43)	125 (47)	94 (54)	1778 (56)	4196 (55)	3036 (54)
Restricted, but ambulatory	6438 (38)	59 (41)	112 (42)	63 (36)	1174 (37)	2876 (38)	2154 (38)
Ambulatory, no hard work	840 (5.0)	15 (10)	19 (7)	12 (7)	140 (4)	364 (5)	290 (5)
Bed half day or completely disabled	395 (2.3)	9 (6)	11 (4)	5 (3)	69 (2)	174 (2)	127 (2)

*Note*: Data are *n* (%) or median (IQR). Overall column shaded grey.

* Stoma at time of survey completion.

### Quality of life

Two ad hoc questions evaluating whether bowel function affected patients' QoL and overall QoL are presented in Table 2. Additional ad hoc questions have been reported in the cohort profile [16]. The proportional distribution of responses to the question ‘Does your bowel function affect your quality of life?’ across the six management groups is presented in Figure 1.

**TABLE 2:** Ad hoc questions evaluating bowel function and overall quality of life (QoL), stratified according to stoma status.

	Overall 19,244	Emergency resection		Elective resection		Lap. Lavage	Inpatient	Outpatient	Diverticulosis
Stoma		Yes	No	Yes	No	–	–	–	–
*n* (%)		49 (0.3)	177 (1)	29 (0.2)	275 (1)	190 (1)	3528 (18)	8663 (45)	6393 (33)
Bowel function affects QoL									
Not at all	7751 (47)	11 (25)	34 (35)	4 (14)	44 (19)	68 (41)	1104 (36)	3438 (46)	3048 (56)
A little	5845 (35)	12 (27)	31 (32)	11 (38)	81 (35)	58 (35)	1277 (41)	2679 (36)	1696 (31)
Quite a bit	2190 (13)	11 (25)	20 (21)	9 (31)	58 (25)	28 (17)	531 (17)	983 (13)	550 (10)
Extremely	801 (5)	10 (23)	11 (11)	5 (17)	48 (21)	13 (8)	196 (6)	336 (5)	182 (3)
Overall QoL									
Very good	6067 (37)	12 (27)	29 (30)	23 (79)^a^	45 (19)	54 (32)	981 (32)	2695 (36)	2250 (41)
Good	8796 (53)	17 (39)	49 (51)		133 (58)	93 (56)	1732 (56)	3995 (54)	2755 (50)
Bad/very bad	1722 (11)	15 (34)	18 (19)	6 (20)	53 (23)	20 (12)	394 (13)	746 (10)	470 (9)

*Note*: Overall column shaded grey.

^a^ Due to a small number of observations, the categories ‘very good’ and ‘good’ were combined.

**FIGURE 1:**
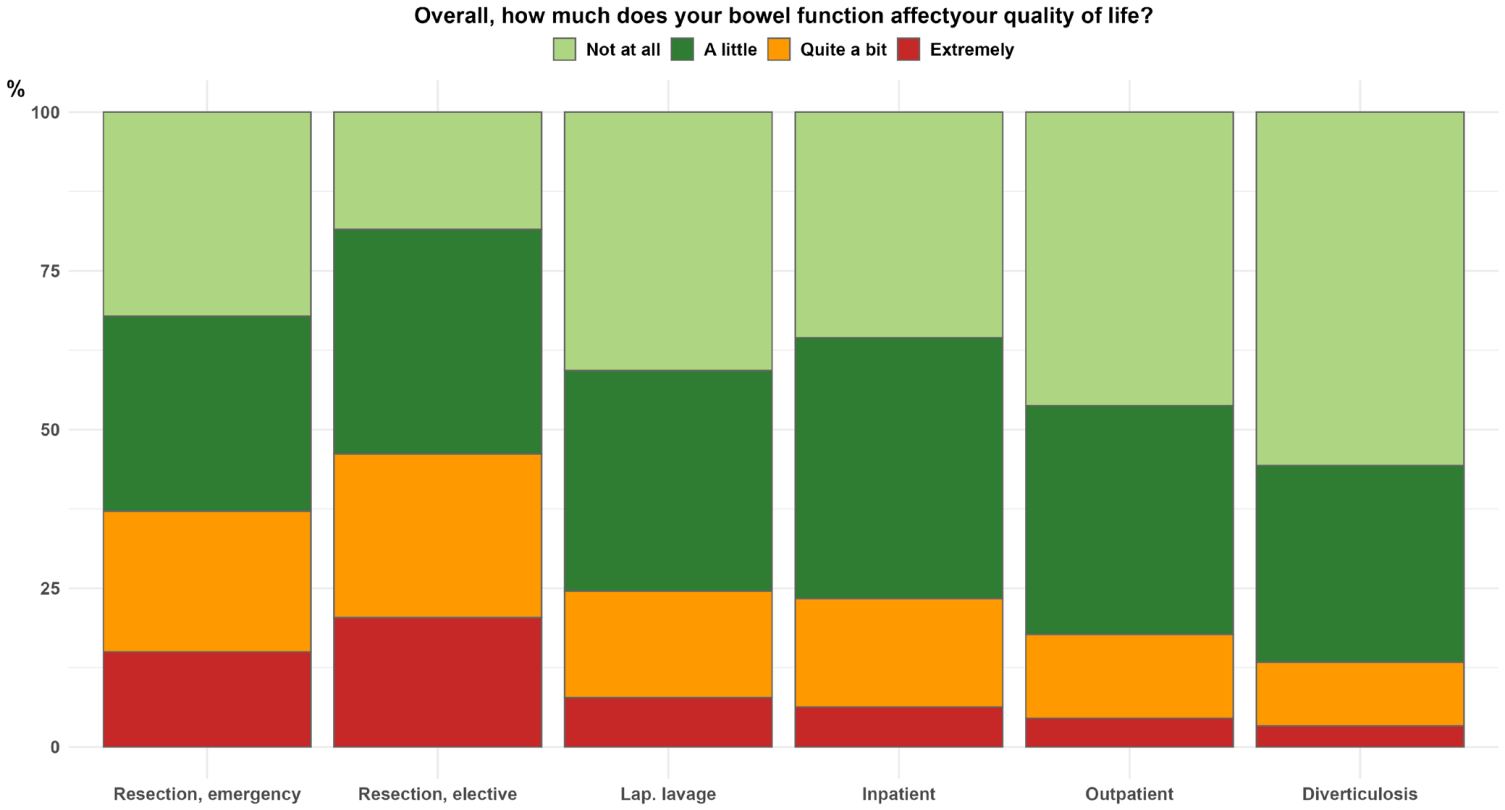
Proportional distribution of responses to the question ‘Overall, how much does your bowel function affect your quality of life?’ across the management groups. Bars show the proportion of respondents in each response category within each management group.

#### Generic quality of life: EQ VAS score

Median EQ‐VAS scores were 80 overall and, for the management groups, 74 (IQR: 50–87) among patients who had undergone emergency colonic resection, 75 (IQR: 53–85) among those who had undergone elective colonic resection, 80 (IQR: 62–90) among those who had undergone laparoscopic peritoneal lavage, 80 (IQR: 65–90) among inpatients, 80 (IQR: 66–90) among outpatients, and 81 (IQR: 69–91) among individuals with diverticulosis recorded solely as a secondary diagnosis.

#### Disease‐specific quality of life: DV‐QoL

The entire DV‐QoL instrument was completed by 17,056 respondents (89% of all). Median DV‐QoL total scores were 2.5 (IQR: 2.0–3.6) overall and ranged from 3.9 among those who had undergone elective colonic resection to 2.2 among individuals in the diverticulosis group (Figure 2). Scores of the four DV‐QoL domains across groups are reported in Table 3 and illustrated in the Figure S1. Patients with resection or inpatient contact reported high *physical symptom* burden, with median scores ≥3.2. The highest median scores (score: 4) were reported by the elective resection patients in the domains of *concerns* and *feelings* (Table 3).

**FIGURE 2:**
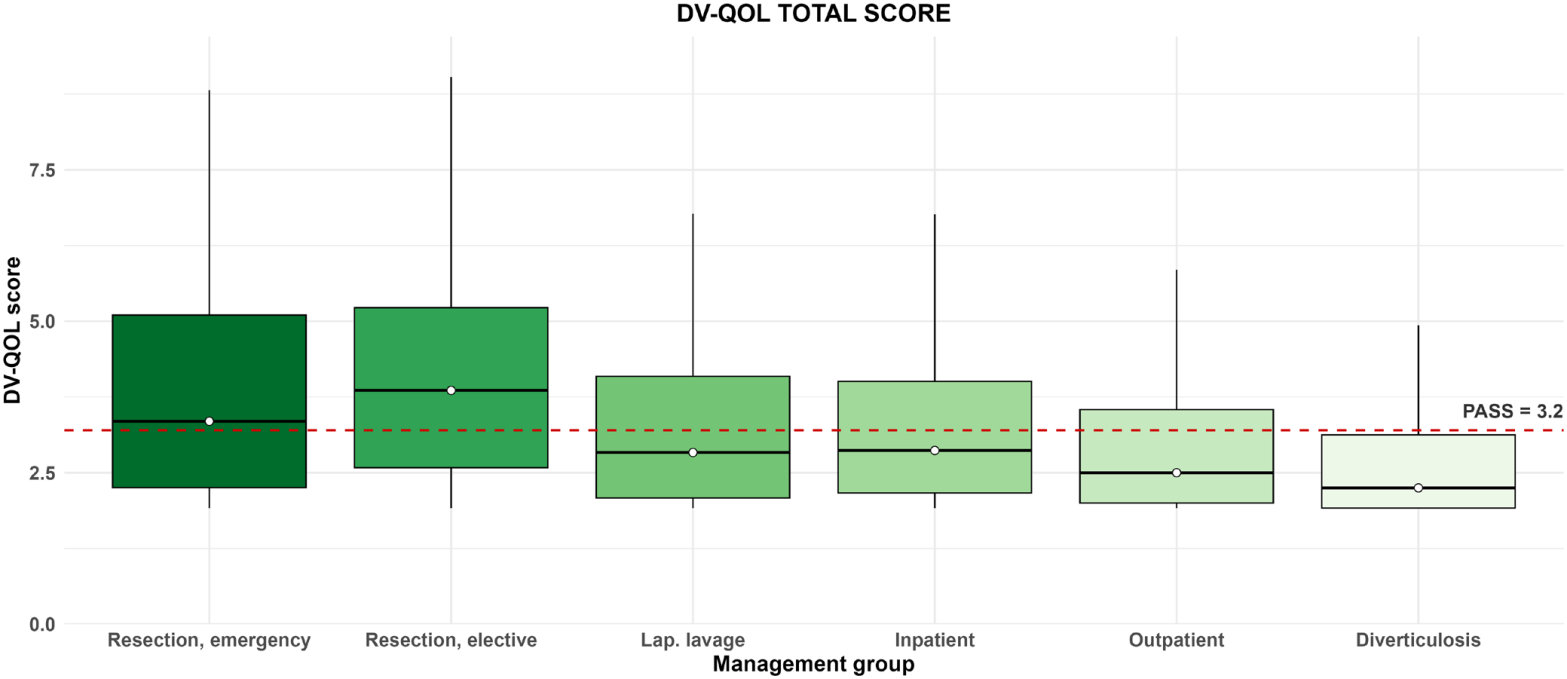
DV‐QOL total score across management groups. 0 best score, 10 worst. Patient‐acceptable symptom state (PASS) has previously been established to 3.2; hence, DV‐QOL total score ≥3.2 indicates an unacceptable symptom state.

**TABLE 3:** DV‐QoL scores.

Score (0–10)	Overall 19,244	Emergency resection 166 (1%)	Elective resection 304 (2%)	Lap. Lavage 190 (1%)	Inpatient 3528 (18%)	Outpatient 8663 (45%)	Diverticulosis 6393 (33%)	*p*‐value
Symptoms	2.7 (1.7–4.0)	3.3 (2.0–5.0)	3.7 (2.3–5.3)	3.0 (2.0–4.3)	3.3 (2.0–4.3)	2.7 (1.7–4.0)	2.3 (1.7–3.7)	<0.001
Concerns	2.0 (2.0–4.0)	3.3 (2.0–5.3)	4.0 (2.0–5.3)	2.7 (2.0–4.0)	2.7 (2.0–4.0)	2.0 (2.0–4.0)	2.0 (2.0–3.3)	<0.001
Feelings	2.0 (2.0–3.5)	3.0 (2.0–5.5)	4.0 (2.0–5.5)	2.5 (2.0–4.5)	3.0 (2.0–4.0)	2.0 (2.0–3.5)	2.0 (2.0–3.0)	<0.001
Behaviour	2.0 (2.0–3.2)	3.2 (2.0–5.2)	3.6 (2.0–5.2)	2.0 (2.0–4.0)	2.4 (2.0–4.0)	2.0 (2.0–3.2)	2.0 (2.0–2.8)	<0.001
Total DVQoL	2.5 (2.0–3.6)	3.3 (2.3–5.1)	3.9 (2.6–5.2)	2.8 (2.1–4.1)	2.9 (2.2–4.0)	2.5 (2.0–3.5)	2.2 (1.9–3.1)	<0.001
Patient‐acceptable symptom state (score <3.2)								
Acceptable	11,647 (68)	68 (47)	105 (38)	102 (58)	1869 (58)	5238 (68)	4265 (76)	<0.001
Unacceptable	5409 (32)	78 (53)	168 (62)	75 (42)	1344 (42)	2417 (32)	1327 (24)	

*Note*: The scale is from 0 (best) to 10 (worst). A total score ≥3.2 indicates QoL‐impacting disease. Colour intensity represents the magnitude of the DV‐QOL score, with darker shading indicating greater symptom burden.

#### Patient‐acceptable symptom state (PASS)

An unacceptable symptom state (i.e. total DV‐QoL score ≥3.2) was reported by 32% of respondents overall, ranging from to 62% in the elective resection group to 24% in the diverticulosis group. The distribution of PASS is presented in Table 3 and Figure 3.

**FIGURE 3:**
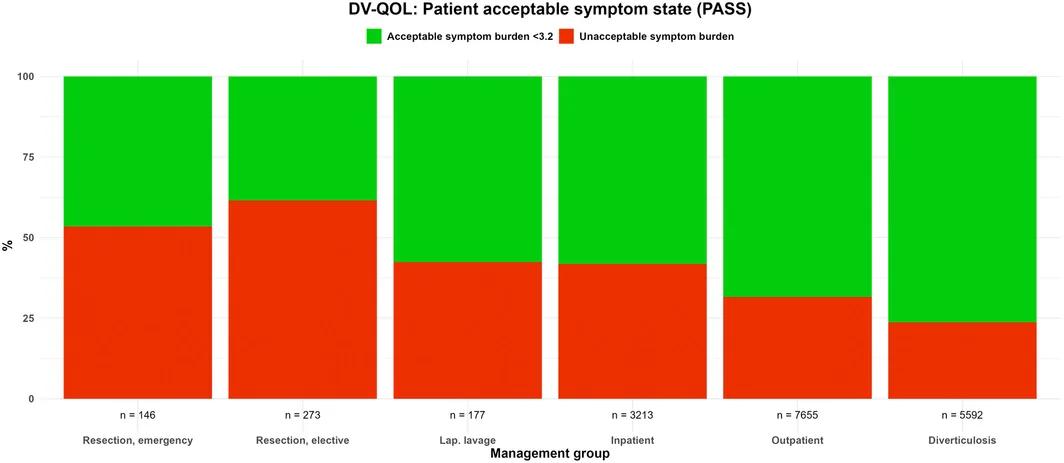
Proportion of respondents in the different management groups with an acceptable or unacceptable symptom state evaluated by the DV‐QOL instrument.

The proportion of patients reporting an unacceptable symptom state according to demographic and clinical characteristics is presented in Table 4. An unacceptable symptom state was reported by more than half of patients younger than 50 years of age, as well as patients who had previously undergone both emergency and elective resection (Table 3).

**TABLE 4:** Univariate and multivariate analysis of patient characteristics associated with QoL‐impacting disease (i.e. PASS ≥3.2).

Characteristic		Complete answer of the DV‐QOL		DV‐QOL score above PASS		Univariate regression		Multivariate regression	
		*N*	%	*N*	%^a^	OR	95% CI	OR	95% CI
Sex	Female	8801	52	3415	39	Ref			
	Male	8255	48	1994	24	0.50	0.47–0.54	0.52	0.49–0.56
Age	<50 years	580	3	315	54	1.82	1.52–2.19	1.80	1.49–2.17
	50–59	2412	14	952	39	Ref			
	60–69	4983	29	1701	34	0.79	0.72–0.88	0.83	0.75–0.93
	70–79	6591	39	1738	26	0.55	0.50–0.61	0.61	0.55–0.67
	≥80	2490	15	703	28	0.60	0.54–0.68	0.67	0.59–0.76
Management group	Diverticulosis	5592	33	1327	24	Ref			
	Outpatient	7655	45	2417	32	1.48	1.37–1.60	1.40	1.30–1.52
	Inpatient	3213	19	1344	42	2.31	2.11–2.54	1.83	1.66–2.01
	Lap. lavage	177	1	75	42	2.36	1.74–3.20	1.84	1.34–2.51
	Elective resection	273	2	168	62	5.14	4.01–6.63	4.24	3.29–5.50
	Emergency resection	146	1	78	53	3.69	2.65–5.14	3.19	2.28–4.48
Charlson comorbidity index score	0	9931	58	3138	32	Ref			
	1–2	5012	29	1605	32	1.02	0.95–1.10		
	3+	2113	12	666	32	1.00	0.90–1.10		
BMI	<18.5	149	1	53	36	Ref			
	18.5–24.9	5314	32	1639	31	0.81	0.58–1.14		
	25–29.9	6706	41	2014	30	0.78	0.56–1.10		
	≥30	4302	26	1509	35	0.98	0.70–1.38		
Time from diverticulosis diagnosis to survey	<2 years	3197	19	1053	33	Ref			
	2–4 years	4253	25	1311	31	0.91	0.82–1.00		
	4–7 years	4836	28	1464	30	0.88	0.80–0.97		
	≥7 years	4770	28	1581	33	1.01	0.92–1.11		
Hospital contacts^a^ due to diverticulosis before survey	0	5615	37	1333	24	Ref			
	1	7167	42	2201	31	1.42	1.32–1.54		
	2–3	3219	19	1267	39	2.09	1.90–2.29		
	4+	1055	6	608	58	4.37	3.81–5.01		
Self‐reported performance status	Fully active	9154	55	2260	25	Ref			
	Restricted, but ambulatory	6295	38	2432	39	1.92	1.79–2.06		
	Ambulatory, no hard work	807	5	350	43	2.34	2.02–2.71		
	No hard work or completely disabled	372	2	208	56	3.87	3.14–4.78		

^a^ Proportion of responders with the characteristic who had PASS ≥3.2.

#### Specific items in DV‐QoL instrument

Responses to all specific items in the DV‐QoL instrument across the management groups are presented in Figures S2. Overall, responses in the resection groups were largely similar and demonstrated the highest symptom burden across all domains. Respondents in the diverticulosis group reported minimal impact of diverticular disease; nonetheless, a small proportion of the group reported a high symptom burden. Overall, outpatients, inpatients and the laparoscopic lavage group reported intermediate levels of impairment.

##### Symptoms

Within the *physical symptoms'* domain, approximately 25% of the resected patients reported incomplete bowel movements and abdominal pain on at least six of the preceding 14 days. In contrast, these symptoms were reported at the same frequency by approximately 10% in the remaining management groups. Feeling bloated at least in six of the last 14 days was reported by approximately 25% in the resection groups, 20% among inpatients and laparoscopic lavage patients, and 10% among outpatients and respondents with diverticulosis.

##### Concerns

*Concern* regarding diverticulitis flare‐up was low among respondents without previous resection, with ≥84% reporting minor or no concerns. On the contrary, approximately 33% of respondents with previous resection reported concerns about recurrent flare‐ups.

##### Feelings

*Feelings* of frustration, anxiety and irritation because of diverticulitis (sometimes/often/always in the preceding 2 weeks) were reported by more than 33% in the resection groups and by more than 25% among the inpatient and laparoscopic lavage groups. Corresponding proportions were less than 20% among outpatients and less than 16% in the diverticulosis group. Overall, only very few respondents reported feeling looked down upon because of their diverticulitis.

##### Behaviour

Among patients with previous resection, approximately 25% reported avoiding social events and/or missing work and other important obligations because of their diverticulitis (sometimes/often/always in the preceding 2 weeks). In contrast, fewer than 10% of respondents in the remaining management groups reported similar behavioural impact.

### Characteristics associated with an unacceptable symptom state

Female sex, younger age, increasing number of hospital contacts, and reduced self‐rated performance status were associated with an unacceptable symptom state, whereas the CCI score, BMI, and time from diagnosis of diverticulosis to survey showed limited association (Table 4).

Compared with the diverticulosis group, all other groups remained associated with higher odds of an unacceptable symptom state after adjustment for age and sex. However, the odds ratios were lower than in the univariate analyses. In the multivariable model, the strongest association was observed for elective colonic resection followed by emergency colonic resection (Table 4).

## DISCUSSION

In this large population‐based cohort of individuals with diverticulosis, reduced quality of life and substantial symptom burden were observed across the spectrum of diverticular disease. Though the greatest impairment was reported among patients with previous emergency or elective resection, impaired disease‐specific QoL was also evident among patients without previous hospitalisation or surgery. Importantly, psychosocial consequences, including concerns regarding recurrent flare‐up, frustration, anxiety and social avoidance, remained common several years after surgical treatment.

This study provides a more nuanced understanding of how diverticulosis affects patients' QoL and everyday life across the full spectrum of disease presentation. Previous studies evaluating QoL in subjects with diverticulosis have predominantly focused on patients admitted with acute diverticulitis or selected surgical cohorts following elective resection [5, 7, 8, 9, 10, 11, 12, 13, 14, 24, 25, 26, 27]. Consequently, knowledge regarding the broader impact of diverticulosis in unselected populations has remained limited. Additionally, previous studies have primarily evaluated generic QoL or gastrointestinal function with patient‐reported outcome measures not specifically validated for subjects with diverticulosis. The present findings suggest that the burden of diverticular disease extends beyond acute inflammatory episodes and traditional clinical outcomes. Although respondents with uncomplicated diverticulosis generally reported lower symptom burden than patients with previous hospitalisation or surgery, a considerable proportion still reported unacceptable symptom burden and reduced disease‐specific QoL. This finding is noteworthy, as individuals without previous hospitalisation might be expected to report better QoL. However, the absence of hospitalisation does not necessarily indicate an absence of symptoms or disease‐related concerns. This population has received limited attention in previous research, despite representing a potentially substantial patient population in primary healthcare, where persistent symptoms and their impact on QoL may otherwise remain unrecognised.

Patients with previous resection reported the highest symptom burden across multiple domains of the DV‐QoL instrument, including *concerns*, *feelings* and *behavioural* impact. Notably, these impairments were reported despite a follow‐up of several years. These findings reflect that surgical treatment does not necessarily eliminate psychosocial distress. The findings support the need for careful preoperative counselling and comprehensive long‐term follow‐up strategies addressing both physical and psychosocial outcomes after surgical treatment for diverticular disease. A high *physical symptom* burden was reported by all four groups that had been hospitalised, highlighting that long‐term symptoms are not limited to surgically treated patients.

Female sex, younger age, and impaired self‐rated performance status were factors associated with an unacceptable symptom state. These findings suggest that long‐term patient experience in diverticular disease may be influenced not only by clinical manifestations but also by demographic and psychosocial factors. The strong association between younger age and unacceptable symptom burden is important to notice since hospital admissions for diverticular disease have increased by up to 132% in individuals aged 40–49 years during recent decades [28]. Similar findings have been reported previously, with younger age identified as an independent predictor of impaired disease‐specific QoL among patients with previous acute diverticulitis [23]. Younger individuals may experience greater disruption of occupational, social and family‐related activities, potentially increasing the perceived burden of disease.

The number of previous hospital contacts due to diverticular disease was also strongly associated with an unacceptable symptom state, with a progressive increase in odds according to the number of contacts. This may reflect greater disease burden, recurrent symptoms, or complications. Repeated healthcare encounters may also influence patients' perceptions of their disease and QoL. However, given the cross‐sectional design of this study, it is not possible to determine whether repeated healthcare contact itself influenced QoL or primarily reflected underlying disease severity.

The finding of substantial symptom burden among patients who had undergone elective resection warrants careful interpretation. Although these patients reported the poorest disease‐specific QoL in the present study, the cross‐sectional design precludes assessment of changes in QoL following surgery. Consequently, it cannot be determined how surgery impacted QoL. Patients selected for elective resection are likely to represent a subgroup with particularly severe symptoms before surgery, which may partly explain the observed findings. Importantly, randomised trials have demonstrated significant improvements in QoL following elective resection compared with conservative management in selected patients with recurrent or persistent diverticular disease [4, 5, 6, 7]. Therefore, the present findings should not be interpreted as evidence that elective surgery adversely affects QoL. Rather, these findings underline that a substantial physical and psychosocial burden may persist in some patients despite surgical treatment and emphasise the need for careful patient selection and realistic preoperative counselling.

The present findings have several potential clinical implications. Current management strategies for diverticular disease primarily focus on preventing complications and recurrent admissions. However, the substantial psychosocial and physical burden identified across management groups suggests that patient‐reported outcomes should play a more central role in long‐term management and follow‐up. In particular, patients with previous surgery, younger individuals, and women may represent groups at increased risk of persistent symptom burden and impaired disease‐specific QoL.

These findings also have implications for future research. Longitudinal studies using validated disease‐specific patient‐reported outcome measures should investigate symptom and QoL trajectories across the full spectrum of diverticular disease, including patients without previous hospitalisation. Such studies may help in evaluating the effectiveness of different management strategies and inform the selection of patients for elective resection by identifying those with persistent symptom burden who may derive the greatest benefit from surgery.

The strengths of this study include the large population‐based cohort, the very high response rate, the linkage to national healthcare registries, and the evaluation of disease‐specific QoL by the DV‐QoL validated in the cohort [17]. Furthermore, the cohort included individuals across the full clinical spectrum of diverticular disease, including patients without previous hospital admission.

Several limitations should be acknowledged. First, the cross‐sectional design precludes drawing conclusions about causality and temporal relationships between diverticular disease, surgical treatment and QoL, as well as the potential contribution of stoma status. Furthermore, the design limits the ability to distinguish symptoms attributable to diverticulosis from symptoms related to other coexisting conditions. Inclusion of a matched control group without diverticulosis would have strengthened the ability to assess the extent to which the reported symptom burden was specifically associated with diverticulosis.

Second, assessment of QoL and symptom burden relied on self‐reported measures and may therefore be subject to recall bias. However, the DV‐QoL instrument uses a relatively short recall period of 2 weeks, which may reduce this risk. Finally, missing data represented a limitation, although analyses were performed using available responses.

## CONCLUSIONS

Diverticular disease is associated with a substantial and sustained burden that extends beyond acute clinical episodes and is not fully captured by traditional disease classifications. Patient‐reported symptom burden appears to be influenced not only by management strategies but also strongly by demographic and functional characteristics. These findings highlight the need for a patient‐centred approach to diverticular disease management that incorporates systematic assessment of long‐term QoL and psychosocial outcomes, with particular attention to patient subgroups at higher risk of persistent impairment.

## AUTHOR CONTRIBUTIONS

**Helene R. Dalby:** Conceptualization; investigation; funding acquisition; writing – original draft; methodology; validation; visualization; writing – review and editing; software; formal analysis; project administration; data curation; resources. **Rune Erichsen:** Supervision; conceptualization; writing – review and editing; methodology; validation. **Katrine J. Emmertsen:** Supervision; conceptualization; funding acquisition; writing – review and editing; methodology; validation.

## FUNDING

Funded by The Novo Nordisk Foundation (Grant ref. no. NNF21OC0071125).

## CONFLICT OF INTEREST STATEMENT

The authors report no conflicts of interest in this work.

## ETHICS STATEMENT

The study was registered at the Danish Data Protection Agency in the Central Denmark Region (record no. 1‐16‐02‐61‐22), and the disclosure of patient information was approved by the Central Denmark Region (record no. 1‐45‐70‐19‐22).

## INFORMED CONSENT

Written informed consent was obtained from all patients.

## Supporting information


Appendix S1.

## Data Availability

The data that support the findings of this study are available on request from the corresponding author. The data are not publicly available due to privacy and ethical restrictions.
