# Supplementary material for: Quality of life in diverticulosis across different clinical manifestations: Findings from the DIVIPACT cohort including 19,244 patients
Source: Colorectal Dis. 2026 Nov 12;28(11):e70681. doi: 10.1111/codi.70681 (PMC13756562; doi:10.1111/codi.70681)
Supplement: Supplementary file 1 [file CODI-28-0-s001.docx]

### Supplementary figures

Figure S-1: DV-QOL scores of all four domains (A: symptoms, B: concerns, C: feelings, D: behaviour) across management groups. 0 best score, 10 worst. Patient-acceptable symptom state (PASS) has previously been established to 3.2, hence, DV-QOL total score ≥3.2 indicates an unacceptable symptom state.

A)

B)

C)

D)

Figure S-2: DV-QOL specific items of all four domains (symptoms, concerns, feelings, behaviour) across management groups. Numbers in the bars are proportions (%).
